# Real-Time Monitoring of Doxorubicin Release from Hybrid Nanoporous Anodic Alumina Structures

**DOI:** 10.3390/s21237819

**Published:** 2021-11-24

**Authors:** Pankaj Kapruwan, Josep Ferré-Borrull, Lluis F. Marsal

**Affiliations:** Departament d’ Enginyeria Electrònica, Elèctrica i Automàtica, Universitat Rovira i Virgili, Avinguda Països Catalans 26, 43007 Tarragona, Spain; pankaj.kapruwan@urv.cat (P.K.); josep.ferre@urv.cat (J.F.-B.)

**Keywords:** hybrid nanoporous anodic alumina gradient-index filters, doxorubicin, Real-Time Monitoring, reflectance spectroscopy

## Abstract

This work demonstrates an advanced approach to fabricate Hybrid nanoporous anodic alumina gradient-index filters (Hy-NAA-GIFs) through a heterogeneous anodization process combining sinusoidal current-density anodization and constant potential anodization. As a result, the hybrid structure obtained reveals a single photonic stopband (PSB), which falls within the absorption region of the drug molecule and the intensity of the spectrum that are far from such absorption range. The prepared structures were loaded with the doxorubicin (DOX) drug through the drop-casting method, which allows for evaluating the maximum reflectance of the relative height of the PSB with the average reflectance of the spectrum intensity. Thereafter, this property has been applied in a flow cell setup connected to a reflectance spectrophotometer where different drug-loaded samples were placed to study the behavior and kinetics of the drug release in real-time by varying two parameters, i.e., different pore length and flow rates. As such, obtained results were analyzed with a model that includes a sum of two inverted exponential decay functions with two different characteristic time releases. Overall, this study opens up several possibilities for the Hy-NAA-GIFs to study the drug kinetics from nanoporous structures.

## 1. Introduction

The emergence of nanoporous materials fabricated through electrochemical anodization in the past decade has led to the utilization of these materials in a plethora of applications ranging from biosensing [1,2,3], photocatalysis [4], to drug delivery [5,6,7,8,9,10]. The innovative ways to obtain advanced nanoporous structures have been explored, which is of utmost importance to control light-matter interactions at the nanoscale regime.

Masuda et al. achieved the breakthrough by introducing a two-step electrochemical anodization process leading to the formation of self-ordered nanoporous anodic alumina (NAA) that extended the capability of this material to prepare advanced nanostructures [11]. The process developed was cost-effective with the ease of fabricating multiple batches of NAA at a laboratory scale. In addition, NAA provides mechanical robustness, stable optical signals and versatile chemistry suitable for multiple applications [12,13,14,15,16,17,18]. The optical properties of NAA can be modulated by varying its refractive index in several ways to obtain advanced structures capable of undergoing optical phenomenon’s (absorption, reflection, emission, and transmission). Numerous anodization strategies such as sinusoidal [19], saw-tooth [20], step-wise [4], and gaussian [21] have been performed in the past, leading to the generation of advanced photonic structures such as microcavities [4], gradient-index filters (GIFs) [2,22], distributed Bragg reflectors [23], and bandpass filters [24], etc. Amongst the structures mentioned above, GIFs display a unique class of photonic structures wherein the periodic variation of the effective refractive index occurs due to the sinusoidal anodization process. As a result of smooth variation of the effective refractive index, GIFs show a well-defined and narrow photonic stopband (PSB) that is highly sensitive to the changes in the effective medium, thus providing an excellent opportunity to study the interaction of several biomolecules. In this regard, GIFs based on nanoporous anodic alumina demonstrate a particular class of optical materials capable of light modulation within the spectral region (Ultra-violet-Vis to Infra-red).

Recent advances in the local administration of drug molecules have involved a range of nanoporous materials ranging from nanoporous silicon, nanoporous anodic titania, and anodic alumina due to their excellent drug loading capacity, stability, and biocompatibility [25,26,27,28,29,30,31]. Alumina (Al_2_O_3_) is considered to be a highly insoluble and nontoxic material used in several orthopedic and dental treatments [32]. Usually, the biocompatibility of structures made up of alumina remains one of the top priorities that should be fulfilled with precision. This has already been achieved in the past through several in vitro studies of several biomolecules with NAA structures [33,34,35]. In addition, La Flamme et al. also performed an in vivo study and investigated the effect of polyethylene glycol modified and bare NAA surfaces to observe inflammation and fibrous tissue growth [36]. The results obtained were found to be non-toxic with a short period of inflammation that can be minimized by polyethylene glycol.

The local administration can be helpful in many cases as it leads to controlled drug release directly at the targeted site. Usually, the drug release from these nanoporous structures is calculated through the in vitro collection of the sample at pre-fixed time intervals under static conditions. However, these static systems fail to imitate the dynamic behavior of the drug release. When the releasing medium gets filled with the drug molecules after a specific time interval, the concentration gradient between the substrate (NAA in this case), and the medium decreases, which directly affects the drug release rate, thus resulting in an imprecise estimation [5,37,38]. Since the fluids inside the body are always in dynamic motion, it is crucial to understand the kinetics and mechanisms involved in real-time to reveal the true nature of drug release from these nanoporous structures.

To overcome the restrictions mentioned above, in this study, we have prepared a hybrid nanoporous anodic alumina gradient-index filters (Hy-NAA-GIFs) structure capable of providing a PSB to be analyzed and, at the same time, capable of holding the drug molecules effectively. For this purpose, Doxorubicin (DOX), an anti-cancer drug was chosen due to its wide usage in chemotherapy [39,40,41,42]. The prepared structure shows a single PSB within the absorption region of the drug molecule while the intensity of the spectrum obtained far from such absorption is used as a reference. Once designed, it was filled with DOX through the drop-casting method where a drop of drug solution is added to the sample and air-dried. The loaded structures were tested in a flow cell setup connected to reflectance spectroscopy setup to demonstrate the release in real-time by varying different flow rates and pore lengths. The evolution of the reflectance spectrum with time was analyzed by applying a model that consists of a sum of two inverted exponential decay functions revealing a diffusion-controlled release from the structures.

## 2. Materials and Methods

### 2.1. Materials

Aluminium foils (thickness 0.5 mm, purity 99.999%) were obtained through Goodfellow Ltd. (Cambridge, UK), ethanol absolute (C_2_H_5_OH, 99.0%, ACS reagent), whereas acetone ((CH_3_)_2_CO, ACS Basic), perchloric acid (HClO_4_, 70% ACS), oxalic acid (H_2_C_2_O_4_, C.N. 144-62-7, M_w_ = 90.03 g/mol), hydrochloric acid (HCl, 37%, C.N.7647-01-0), copper chloride (CuCl_2_, C.N.-10125-13-0, >99%), Doxorubicin hydrochloride (DOX, C.N. 25316-40-9), phosphate buffer saline (PBS, bioperformance, pH-7.4) were purchased from Sigma Aldrich (Merck, Darmstadt, Germany). Double deionized water (DI) (18.6 MΩ, PURELAB Option Q) was used for all the solutions unless otherwise specified.

### 2.2. Fabrication of Hy-NAA-GIFs

Hy-NAA-GIFs samples were prepared by combining sinusoidal current anodization and constant potential anodization in a one-step electrochemical process. In brief, freshly cut aluminium pieces were treated with acetone, ethanol, and water to remove all the impurities that reside on the surface. This step was followed by the electropolishing step where the aluminium (anode) and platinum wire (cathode) were placed in a mixture of 4:1 *v*/*v* of ethanol: perchloric acid at 20 V for 4 min by switching the direction of stirring every 60 s to obtain the smooth surface. To prepare the modulated structure on the top part of the Hy-NAA-GIFs, a sinusoidal current density profile variable with time was applied in an electrochemical cell containing 0.3 M oxalic acid at 5.5 °C under controlled stirring. The sinusoidal parameters applied in this study were obtained with *j*_0_ = 2.6 mA/cm^2^, *j*_1_ = 1.3 mA/cm^2^; *T* = 133 s, N = 150 periods. This step was followed by constant potential anodization profile to grow the straight channels by controlling a total employed charge with a voltage of 40 V to obtain different lengths of the bottom part of the structure. Following this procedure, three different sets of samples were prepared and denoted as short (24.6 µm), medium (28.9 µm), and long (84.4 µm) Hy-NAA-GIFs samples.

The sinusoidal current density anodization profile was applied with a Keithley 2400 source meter, which was controlled by LabVIEW^®^ software program based on the following dependence of current density with time Equation (1):

(1) $$j\left(t\right)=j_{0}+j_{1}\cdot \sin\left(\frac{2\pi }{T}t\right)\mathrm{for}\;0\;<\;t\;<\;N\;\cdot T$$

where *j*(*t*) corresponds to the current density at the time *t*, *j*_0_ and *j*_1_ are the average current density and the amplitude of sinusoidal current density variations. *T* represents the time period of the sinusoidal variations and N is the number of cycles applied.

**Figure 1:**
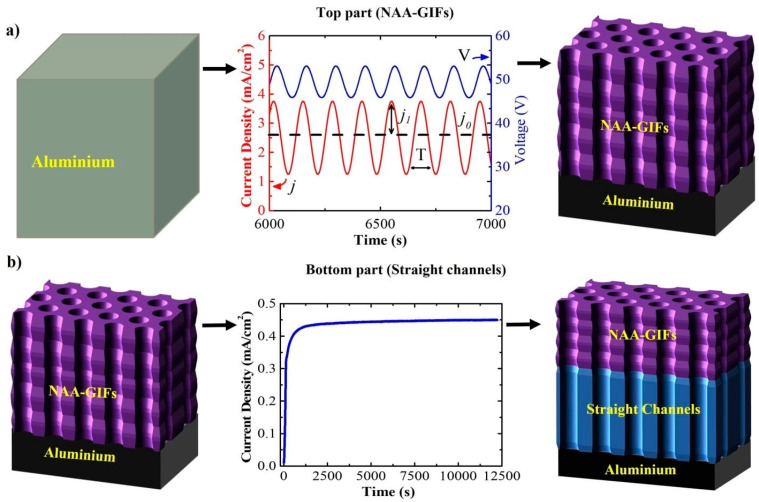
Schematic diagram of the electrochemical approach applied to prepare Hy-NAA-GIFs. (**a**) represents a sinusoidal current density anodization profile *j* and V to obtain the modulated on the top part of the structure., *j*_1_ is the measured cell current density for the amplitude of sinusoidal variations and *j*_0_ represents the offset current density, *T* depicts the time period of the process; (**b**) shows the constant potential anodization process to obtain the straight channels at the bottom.

The structure obtained through the sinusoidal current density anodization leads to the formation of the modulated structure onto the top, while the constant potential anodization applied results in the formation of straight channels at the bottom, thus constructing advanced Hy-NAA-GIFs (Figure 1).

It has been noticed with the previous experiments that the etching of the aluminium from the backside helps in improving the contrast of the PSB in the reflectance spectrum and also allows to see the interferometric color with the naked eye [43]. Therefore, the aluminium layer at the bottom was etched in a solution of HCl (100 mL in 400 mL H_2_O) and CuCl_2_ (13.6 g added to the HCl/H_2_O mixture). This step was followed by a pore widening step, which was performed in 5 wt% H_3_PO_4_ at 35 °C for 10 min for all the samples fabricated.

### 2.3. Doxorubicin Hydrochloride (DOX) Loading

As mentioned in the introduction, this paper utilizes DOX to study the release kinetics in real-time. Inspired by the results obtained in our previous paper, which describes the in-depth filling pattern of a model drug (Rhodamine 6G) inside the NAA-GIFs structure, having two PSBs [43] that relate to the amount of drug filling the pores. In this study, we follow the same protocol for an effective filling and measurement of DOX with UV-VIS. The method utilized is known as drop-casting where a drop of the drug solution is placed on the top of the NAA surface and left to air-dry. The consecutive steps performed, i.e., loading of the drug followed by drying, have been termed as drop/dry cycles.

In brief, DOX solution in ethanol (500 µg/mL) was prepared and 10 µL was dropped onto the surface of the sample followed by air-drying at room temperature. This step was repeated until 6 drop/dry cycles were performed to fill the structure followed by the subsequent measurement by UV-VIS spectroscopy after each step.

### 2.4. Optical Measurements of Hy-NAA-GIFs

The reflectance spectra of Hy-NAA-GIFs were registered with a UV-Visible spectrophotometer (PerkinElmer Lambda 950) attached with a Universal reflectance attachment (URA) at an angle of 8° with a 2 nm resolution. The same setup was also used to measure the drug loading inside the pores. The detailed instrumentation about the flow cell setup has been described in here [43]. In brief, the reflectance spectra of Hy-NAA-GIFs were registered with a light source (halogen) and a USB 2000+ fiber spectrometer (Ocean Optics, Orlando, FL, USA). The samples were placed on the top of the cell made up of acrylic plastic with a channel size of 2 mm. The light was illuminated onto the sample surface through a bundle of optical fibers. The light reflected from the sample was collected by the same fiber and sent to the spectrophotometer for processing. The software was designed to save the spectra after every 10 s while a PBS solution with a pH of 7.4 was flown at a rate of 80 or 140 µL/min through the flow cell.

### 2.5. Structural Characterization

All the structural images containing information about pore diameter, and length were captured by a field-emmission environmental scanning electron microscope (FESEM FEI Quanta 600) at an operating voltage of 20 keV. After obtaining the images, they were processed with an ImageJ software.

## 3. Results and Discussion

### 3.1. Fabrication and Structural Characterization of NAA-GIFs

**Figure 2:**
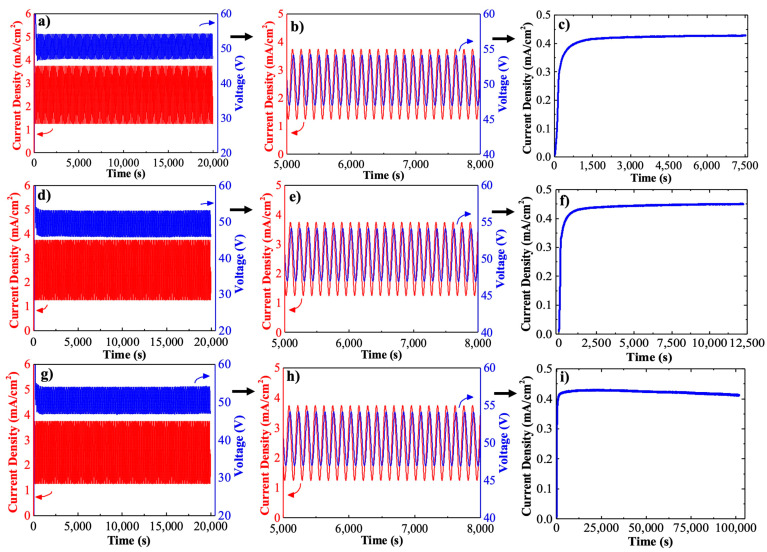
Anodization curves obtained for hy-NAA-GIFs as a result of sinusoidal and constant potential anodization for short (**a**–**c**), medium (**d**–**f**), and long samples (**g**–**i**).

The anodization approach applied in this study helps in reshaping the pore arrangements in Hy-NAA-GIFs through in-depth modulation of pore diameter. Figure 2 demonstrates the profiles obtained by combining the sinusoidal and constant potential anodization processes. Figure 2a,d,g show the complete evolution of the sinusoidal anodization profile for time instants between *t* = 0 s, and *t* = 20,000 s. Figure 2b,e,h depict more detailed profiles of the applied current density and voltage as a function of time where the voltage delay can be observed along with the sinusoidal behavior of the measured voltage in between *t* = 5000 s–8000 s. Finally, Figure 2c,f,i show typical current density-time transients curves during which the growth of the structure shifts from the modulated morphology to the straight channels with a constant anodization voltage of 40 V for all the samples.

It has been shown that when a controlled sinusoidal current is applied, the measured anodization voltage also follows a sinusoidal function with the same period for all the samples. Therefore, these variations in voltage lead to pore diameter modulation inside the structure. For the constant potential anodization method, a noticeable change in the current density-transient curves was observed for all the samples where an exponential increase in current density is observed until a maximum is achieved. Usually, this step is attributed to the rearrangement of pores on the surface for further growth of the structure. In addition, a gradual decrease in current density is observed for long samples which could be possible because of the limitation in the diffusion of the ions deep inside the pores [44,45]. It is apparent from Figure 2a,d,g that there is an initial transient in the measured potential followed by the anodization voltage resembling the sinusoidal behavior of the current sinusoidal variations with a delay. The anodization experiments performed allow a precise modulation of the porosity through sinusoidal variations of the measured anodization voltage varying between 45 V–53 V with an average of 49 V for all the samples. The anodization profiles described above confirmed the fabrication of a Hy-NAA-GIF structure based on the electrochemical conditions provided. 

**Figure 3:**
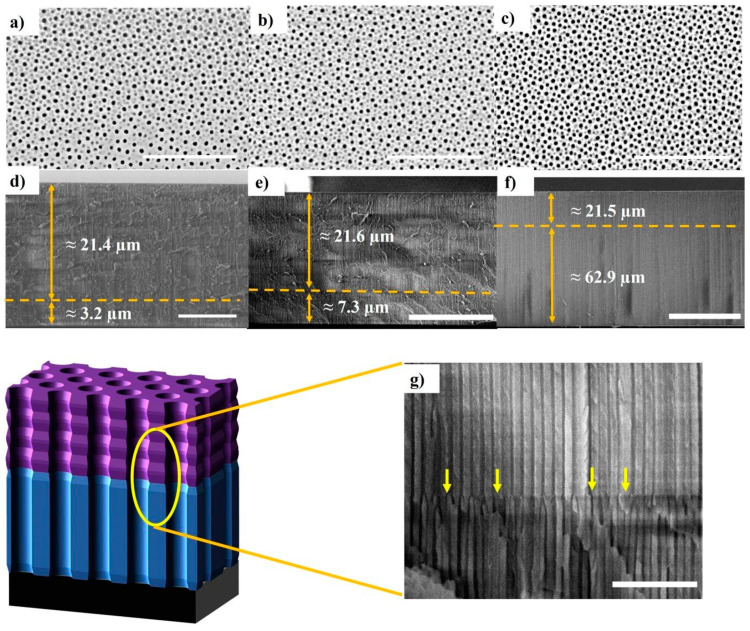
SEM pictures of Hy-NAA-GIF structure obtained with the sinusoidal parameters, *j*_0_ = 2.6 mA/cm^2^, *j*_1_ = 1.3 mA/cm^2^; *T* = 133 s, N = 150 periods and different current-density vs. time parameters. (**a**–**c**) represents top view (Scale bar = 1 µm) and (**d**–**f**) demonstrates cross-section (scale bar = 10, 20 and 50 µm respectively) for the short, medium, and long samples respectively. (**g**) interface between the modulated pore and straight pore parts of the structure caused by the change in the anodization process indicated by arrows.

Figure 3 summarizes the scanning electron microscopy (SEM) images captured to confirm the Hy-NAA-GIFs structure. Figure 3a–c represent the top-view, whereas Figure 3d–f show the cross-sectional image taken for as-produced structure for short, medium, and long samples respectively. Figure 3g represents the magnified version of Figure 3e in graphical form.

The images obtained confirm the fabrication of a Hy-NAA-GIFs structure based on aluminium with a sinusoidal current anodization and a constant potential anodization profile. In Figure 3a–c the top-view SEM images reveal the random arrays of nanopores after the PW process distributed across the surface with an average pore diameter of 39 ± 3 nm and an average interpore distance of 98 ± 16 nm estimated with the ImageJ software. The cross-section analysis was also performed for all the samples, which helps in measuring the total thickness of the prepared samples. For short samples, the modulated structure was found to be around 21.4 µm long while the straight channel was 3.2 µm long (Figure 3d). On the other hand, the medium samples revealed a length of 21.6 µm for the modulated structure and 7.5 µm for the straight (Figure 3e). Similarly, the long samples showed a length of 21.5 µm for the modulated and 62.9 µm for the straight channel (Figure 3f). The obtained images clearly reveal a noticeable change in the structure while switching from sinusoidal to constant potential anodization process (Figure 3g). The measurements obtained for the Hy-NAA-GIFs through digital images depict the correct translation of stacked layers of GIF fabricated through sinusoidal current anodization on the top part followed by the formation of straight channels as a result of constant potential anodization profile at the bottom.

### 3.2. Optical Characterization of Hy-NAA-GIFs

The analysis carried out in our previous study consisting of NAA-GIFs with two PSBs allows for obtaining the correlation between the height of the PSB and the amount of filling species within the structure [43]. In this work, we have designed a structure where the top part consists of GIF while the bottom part contains spectrum intensity. The GIF has a PSB that in the spectrum shows as a high-reflection band, which can be used as a “signal” to estimate the amount of drug within the pores. On the other hand, the bottom part adds length to the different pore structures with a minimal contribution to the spectrum (only Fabry–Perot interferences). For confirming the as-prepared samples to display the characteristics mentioned above, UV-Visible spectra were recorded.

**Figure 4:**
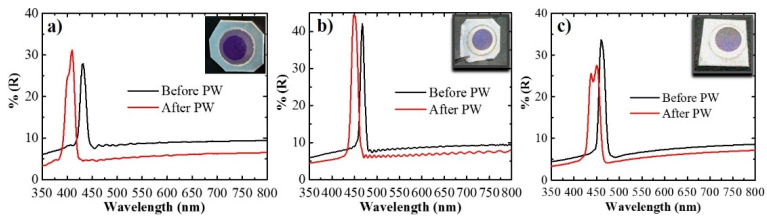
Reflectance spectra (**a**–**c**) of Hy-NAA-GIFs structure obtained for short, medium, and long samples, respectively. Inset shows the digital pictures of as-obtained samples showing the interferometric color.

For the short samples (Figure 4a), the maximum reflectance was recorded at 432 nm with a reflectance of 27.9% before the pore widening (PW), whereas it shifted to 410 nm with a reflectance of 31.1% after the PW process. For the medium samples (Figure 4b), the signal channel was recorded at 468 nm with a reflectance of 42% and 448 nm with a reflectance of 44.3% before and after PW. Finally, for long samples (Figure 4c), before PW, the signal channel appeared at 460 nm with a reflectance of 33.6%, while it moved to 450 nm with a reflectance value of 27.5%. These results confirm the presence of the desired signal channel at the wavelength range of the highest absorption region of the DOX (480–500 nm), in addition to the necessary reference wavelength range required far from this absorption region (700–850 nm). However, for the long samples, an additional side lobe was observed as a result of sharp truncation of the effective refractive index, specifically at the boundaries of the Hy-NAA-GIFs [46].

**Figure 5:**
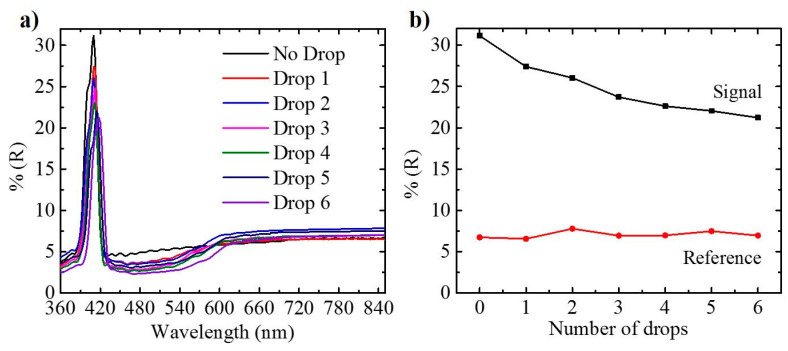
(**a**) Reflection spectra recorded for Hy-NAA-GIF fabricated with *j*_0_ = 2.6 mA/cm^2^, *j*_1_ = 1.3 mA/cm^2^; *T* = 133 s, N = 150 periods for short length sample after each drop/dry cycle, (**b**) illustration of maximum reflectance obtained for the signal (410 nm) and reference wavelength (800 nm) as a function of the number of drops applied.

Figure 5a represents the UV-VIS spectra recorded after each drop/dry cycle and their impact on the signal and reference wavelength ranges. As the first drop of the drug is added to the structure, it leads to a change in the effective medium of the Hy-NAA-GIFs, resulting in a slight decrease in the maximum reflectance value from 31.1% to 27.3% at 410 nm. This value further fluctuates but, overall, a 31.9% decrease in the value of reflectance is observed from 31.1% to 21.2% after the addition of the 6th drop. In addition, minor variations in the reflectance value have also been observed in the non-absorption region of the hybrid structure, which also leads to an assumption of a change in the dielectric constant of the medium of the straight channels due to penetration with the drug molecule.

The main purpose of obtaining the reflectance spectra is to define a metric as the ratio between the maximum reflectance at the PSB range and the average reflectance of a wavelength range far from absorption of DOX for Hy-NAA-GIFs. The trend observed during the infiltration process inside Hy-NAA-GIFs is not homogenous due to an increase in the amount of the drug molecules inside the pores with each drop/dry cycle, hence making it difficult for further molecules to enter. Figure 5b shows a plot demonstrating a decreasing trend of the maximum reflectance of the signal at a wavelength of 410 nm as compared to a slight change in the intensity of spectrum at a wavelength of 800 nm far from the absorption range.

The above-mentioned results successfully confirm that the drop-casting method is one of the effective methods to infiltrate the pores and, simultaneously, can be analyzed for the successful filling by recording the spectra before and after filling using UV-Visible spectroscopy.

### 3.3. Real-Time Monitoring of Drug Using Reflectance Spectroscopy

**Figure 6:**
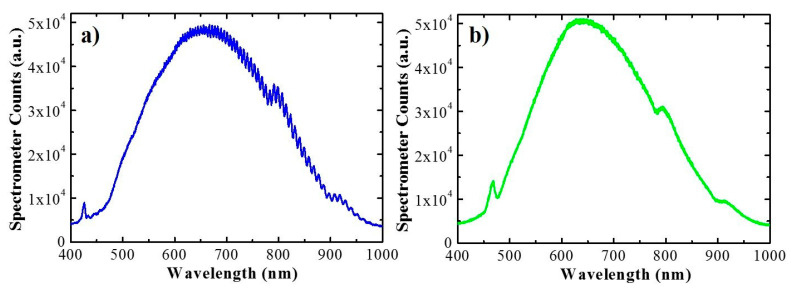
Signal measured by the fiber spectrometer for the short sample (**a**) and long sample (**b**) placed in the flow cell respectively.

Once the Hy-NAA-GIFs was tested successfully for the infiltration of DOX with the drop-casting method, the same protocol was applied without any changes for DOX loading in all the samples for studying the release kinetics in real-time. After the loading, the samples were placed in a flow cell setup that permit the measurement of the sample reflected intensity while PBS flows through the porous structure. Figure 6 shows two of the measured spectra as an example. Figure 6a corresponds to a 25 µm (short) sample in an experiment carried out at a flow rate of 80 µL/min, while Figure 6b corresponds to an 85 µm (long) sample in an experiment with a 140 µL/min.

The magnitude measured by the fiber spectrometer is a number of counts and corresponds to the combined lamp emission spectrum, sample reflectivity and detector sensitivity. The plots show different features, the main being the local maximum between 430 nm and 500 nm caused by the higher reflectance of the gradient-index filter present in the sample. The spectrum for the thinner sample also shows oscillations resulting from the Fabry–Pérot interferences between the two reflections of the spectrometer beam at the extreme interfaces of the porous nanostructure. These oscillations are not present in the long sample as the coherence length of the beam is too small to sustain them.

**Figure 7:**
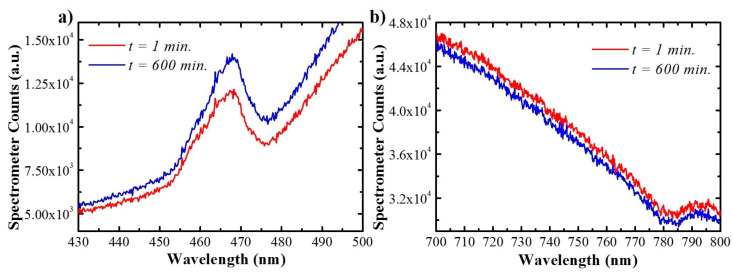
Spectra registered after wetting using reflectance spectroscopy at time instants *t* = 1 and 600 min for wavelength range that include the PSB (**a**), and reference wavelength range (**b**) in Hy-NAA-GIF.

In the release experiment, time *t* is set to *t* = 0 at the instant the PBS is injected in the flow cell. At this instant the wetting of the pores takes place and results in a turbulent flow on the very path of the spectrometer beam that severely interferes with the measurement. After this wetting, the signal stabilizes, and the measured spectra become reliable. The spectra shown in Figure 7 correspond to time instant *t* = 1 min. and *t* = 3.6 *×* 10^4^ s = 600 min. As the drug is released from the nanostructure its concentration within the gradient-index part of the porous structure decreases and produces an increase in the maximum signal of the local maximum. At the same time, since the drug has a very small absorption above 650 nm, the spectra should show very little variation in this range. To illustrate this point, Figure 7 shows the spectra in the wavelength ranges between 430 nm and 500 nm (Figure 7a) and between 700 nm and 800 nm (Figure 7b), at the different release times indicated in the legend.

These two figures show that the maximum signal at the PSB wavelength range shows an increase of 15%, while the signal at the 700–800 nm range shows a decrease of 3%. The increase in the PSB wavelength range confirms that it can be used to estimate the variation in DOX concentration within the gradient-index filter part of the nanopores. Figure 7b also shows that after 10 h of measurement, the signal in the measurement systems has drifted. This drift must be compensated for to achieve reliable estimates of the drug release dynamics (refer to Supplementary Materials, Equations (S1) and (S2), Figure S1).

**Figure 8:**
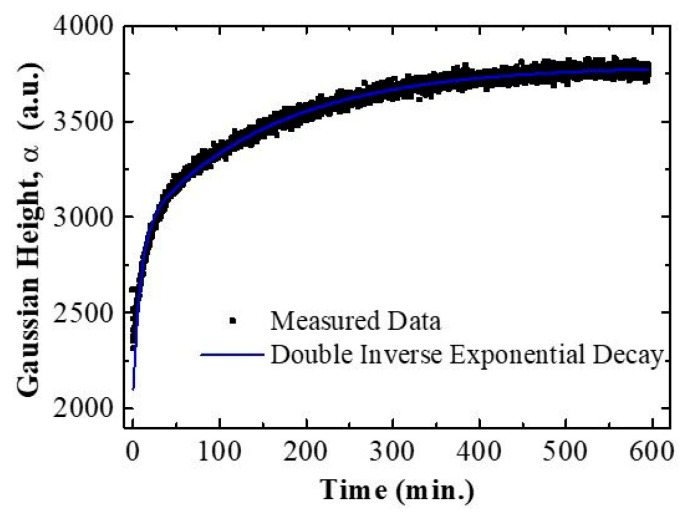
The graph represents the best fitting obtained with the model being applied for the time evolution of the signal for Hy-NAA-GIFs.

In this range, as it can be seen in Figure 7a, the feature corresponding to the reflectance of the sample appears overlapped to a baseline, corresponding to the combined effect of the straight pore structure optical properties and the lamp spectral intensity and detector spectral sensitivity. To properly evaluate the evolution of this maximum reflectance is necessary to distinguish the local maximum related to the gradient-index part of the structure from the baseline. This is accomplished by finding the best fit between the corrected spectrum $S_{i,\mathrm{corrected}}\left(\lambda \right)$ (also refers to Supplementary Materials, Equation (S3) and a model function consisting of the sum of a gaussian component (which accounts for the maximum in reflectance) plus a third-degree polynomial (which accounts for the baseline):

(2) $$G_{i}\left(f\right)=\alpha \cdot \exp\left(-{\left(\frac{f-\beta }{2\sigma }\right)}^{2}\right)+\gamma +\delta \cdot f+\epsilon \cdot f^{2}+\theta \cdot f^{3}\;$$

where f is the inverse of the wavelength (f=1/λ), which is proportional to the photon frequency or energy. In this function, parameter α is the height of the gaussian, β is the center frequency of the gaussian and σ is its spread. Parameters γ, δ, θ, and ϵ are the polynomial coefficients. By using the inverse of wavelengths as the variable of the fitting function, better parameter estimates are obtained. Applying this process to all the measured spectra in a flow cell drug release experiment, the evolution with time (the gaussian height, α) can be obtained. An example of the signal evolution with time is shown in Figure 8. Values for t<0 are not represented as they correspond to the flow cell without the fluid. The first represented point in the plot corresponds to t=1.35 min. Before this time, the signal is distorted by the pore-wetting process. When the signal stabilizes, it shows a clear increasing trend. This trend has a steeper ascent at the beginning and then reduces its growth rate until it plateaus, indicating no more drug is being delivered from the porous nanostructure. All the measurements for all the studied samples show similar behaviors (refer to Supplementary Materials).

To extract information from the evolution of the signal with time, a further analysis by best fit to a model has been applied. Different models have been tested and the one that leads to the best results is the sum of two inverted exponential decays with the same starting point and with two different time constants:

(3) $$\alpha \left(t\right)=\alpha_{0}+\kappa_{1}\left(1-\exp\left(-t/\tau_{1}\;\right)\right)+\kappa_{2}\left(1-\exp\left(-t/\tau_{2}\;\right)\right)$$

In this expression $\alpha_{0}$ is the value of signal at t=0, which is unknown because of the distortion of the spectra during the wetting phase. Parameters $\kappa_{1}$ and $\kappa_{2}$ are the relative weights of the two inverse exponential decay components. Finally, $\tau_{1}$ and $\tau_{2}$ are the time constants of the two exponential decays. Figure 8 includes the result of the best fit of the data to this double inverted exponential decay. A single inverse exponential decay fit was also performed to analyze the time evolution of the signal (refer to Supplementary Materials, Figure S2). It demonstrates that the simpler model cannot reproduce the two regimes of variation present in the data characterized by the two-time constants $\tau_{1}$ and $\tau_{2}$.

**Figure 9:**
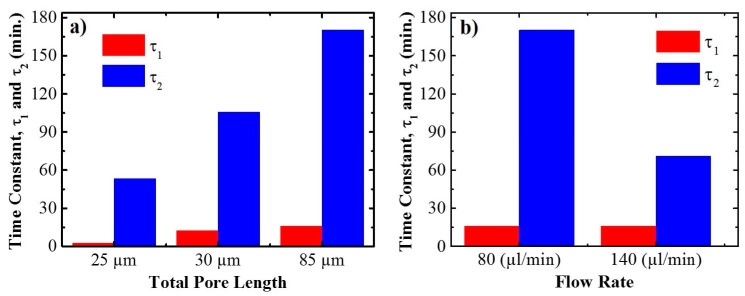
Trend obtained of the two different characteristic time releases of drug from the whole structure as a function of total pore length (**a**), and flow rate (**b**), respectively.

Figure 9 shows a summary of the obtained time constants for the three studied pore lengths (Figure 9a) and the two studied flow rates (Figure 9b). All the release profiles obtained with the different pore lengths and flow rates can be found in Supplementary Materials, Figure S3.

For the three pore lengths, time $\tau_{1}$ is between 2 and 16 min, while time $\tau_{2}$ increases from 53 min for a total pore length of 24.6 µm to 170 min for the total pore length of 84.4 µm. If different flow rates are considered, Figure 9b) shows that time constant $\tau_{1}$ is not influenced by the flow rate, with a value of 16 min for both flow rates. On the other hand, time constant $\tau_{2}$ decreases with increasing flow rate from a value of 170 min for the slowest flow rate (80 µL/min) down to 71 min for the highest rate (140 µL/min).

These results suggest that the two characteristic times correspond to two different mechanisms in the release process. The increase in $\tau_{2}$ with pore length and its decrease with flow rate are both in good agreement with a diffusion-controlled release of the drug from the pores. Instead, $\tau_{1}$ is fairly invariant both for the different pore lengths and the different flow rates. This may be related to a specific effect of the gradient-index filter top part of the pores. This part is the first to be filled by the liquid even before the liquid can reach the bottom, so the release from this part is started earlier and at a faster rate (hence the smaller $\tau_{1}$ value) than the diffusion-controlled release from the structure considered as a whole.

## 4. Conclusions

The research carried out in this work demonstrates the ability of Hy-NAA-GIFs to be used as an advanced photonic structure suitable for analyzing the loading and release of doxorubicin molecules. To demonstrate this, Hy-NAA-GIFs structures were prepared, loaded with the drug solution using the drop-casting method characterized by the measurement with UV-Vis and afterward, monitored in real-time by reflectance spectroscopy to further evaluate the releasing kinetics.

In comparison to the nanostructures with two PSBs prepared in the previous works, herein, nanostructures with only one PSB that can be tuned to fall within the drug absorption region are applied. Instead of a second PSB, in this work the intensity of the spectrum in a wavelength range far from drug absorption acts as a reference. The drop-casting method helps to predict the changes in the filling process by analyzing with UV-V is spectroscopy the relative height of the PSB reflectance maximum with respect to the average reflectance at the reference wavelength range. Once loaded with the drug, these structures are placed in a flow cell setup connected to a reflectance spectroscopy setup and data processing capable of evaluating the drug release in real-time. This permits researchers to obtain an estimate of the amount of drug infiltrating the pores as a function of time. This is then analyzed with a model consisting of a sum of two inverted exponential decay functions with two different characteristic times, $\tau_{1}$ and $\tau_{2}$.

The experiments were conducted by varying two parameters, i.e., different flow rates and pore lengths. Results show that increasing pore length results in a corresponding increase in $\tau_{2}$, without much significant change in $\tau_{1}$. On the other hand, increasing the flow rate also results in a decrease in $\tau_{2}$, while $\tau_{1}$ is unaffected. This reveals the existence of a contribution to release related to the wetting of the top GIF part of the structure characterized by $\tau_{1}$ followed by a diffusion-controlled release from the whole length of the pore characterized by $\tau_{2}$. We believe that the approach developed in this chapter can be further applied to gain information about the loading process of the drug and to further study the drug-releasing behavior in real-time from porous nanostructures.

## Data Availability

Not applicable.

## Supplementary Materials

The following are available online at https://www.mdpi.com/article/10.3390/s21237819/s1, Figure S1: Example of the best fit to obtain the maximum corrected reflectance within the photonic stop band of the structures. Figure S2: best fit of the evolution of the maximum corrected reflectance with time to a single inverse exponential decay model. Figure S3: evolution of the maximum corrected reflectance with time for two different flow rates. Figure S4: evolution of the maximum corrected reflectance with time for three different pore lengths.
