# Supplementary material for: Real-Time Monitoring of Doxorubicin Release from Hybrid Nanoporous Anodic Alumina Structures
Source: Sensors (Basel). 2021 Nov 24;21(23):7819. doi: 10.3390/s21237819 (PMC8659439; doi:10.3390/s21237819)
Supplement: Supplementary file 1 [file sensors-21-07819-s001.zip › sensors-1467625-supplementary.pdf]

Supporting information

Real-time monitoring of Doxorubicin release from Hybrid Nanoporous Anodic Alumina Structures

Pankaj Kapruwan, Josep Ferré-Borrull, Lluis F. Marsal*

Departament d' Enginyeria Electrònica, Elèctrica i Automàtica, Universitat Rovira i Virgili

Avinguda Països Catalans 26, 43007, Tarragona, Spain

*Corresponding author e-mail address: lluis.marsal@urv.cat

Fitting parameters for evaluating the flow cell release

To compensate for the observed drift as well as for the possible noise in the measured spectra, a reference spectrum ($R(\lambda)$) for the 700-800 nm range is determined as the average of all measured spectra with reliable signal in this range in the release experiment. Here, the term reliable signal refers to all those spectra registered after the wetting process of the pores with the stabilized signal. Then, by comparing the spectrum $S_i(\lambda)$, taken at time instant t_i with the reference spectrum in the 700-800 nm range, it is possible to correct the spectrum in all the measured ranges. In this work, we modelled the effect of the fluctuations as an affine transformation. With this, within the 700-800 nm range the measured spectrum should be:

$$S_i(\lambda) = A_i \cdot R(\lambda) + B_i, \;\; 700\,nm < \lambda < 800\,nm \quad \text{(i)}$$

where the constant A_i accounts for a change in scale (that could model a change in beam intensity) while B_i accounts for a change in background signal (that could be attributed to noise). Assuming constants A_i and B_i do not depend on the wavelength, they can be estimated by minimizing a merit function defined as:

$$F(A_i, B_i) = \sum_{\lambda=700\,nm}^{\lambda=800\,nm} |S_i(\lambda) - A_i \cdot R(\lambda) - B_i| \quad \text{(ii)}$$

The A_i and B_i that minimize F permit to obtain the corrected spectrum applying the inverse affine transform:

$$S_{i,corrected}(\lambda) = \frac{S_i(\lambda) - B_i}{A_i}, 430\,nm < \lambda < 500\,nm \quad \text{(iii)}$$

This corrected spectrum is then used to evaluate the height of the local maximum in the PSB wavelength range.

Figure S1 shows an example of the best fit obtained with the function in equation (2) for the same data shown in main manuscript (figure 7) after correction. The value of the gaussian height, indicated in the figure, is used as an estimate of the maximum reflected intensity from the rugate part of the porous nanostructure.

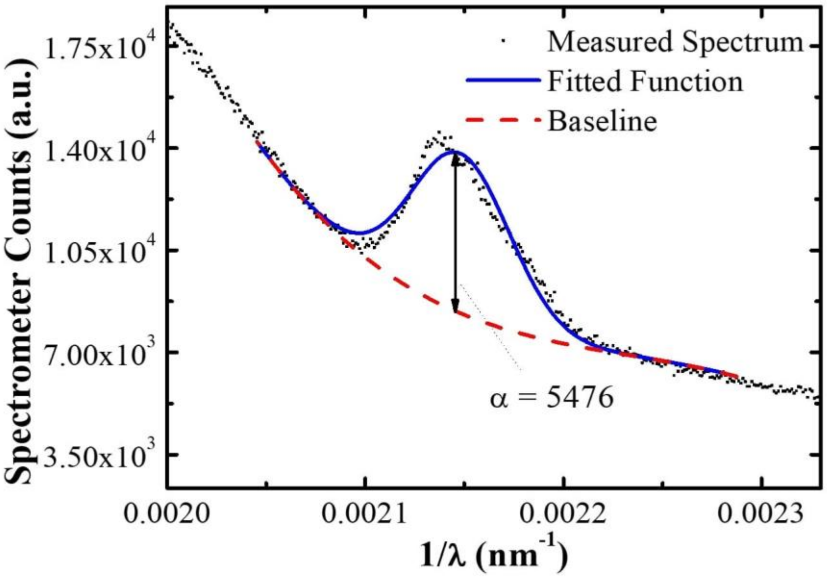

Figure S1: Best fit of function in equation (2) of the main document to the data in figure 7a.

Figure S2 shows the best fit obtained for the evolution of parameter α with time to a model consisting of a single inverted exponential decay. This result shows that the adequate model is the double inverted exponential decay of equation (3) in the main manuscript.

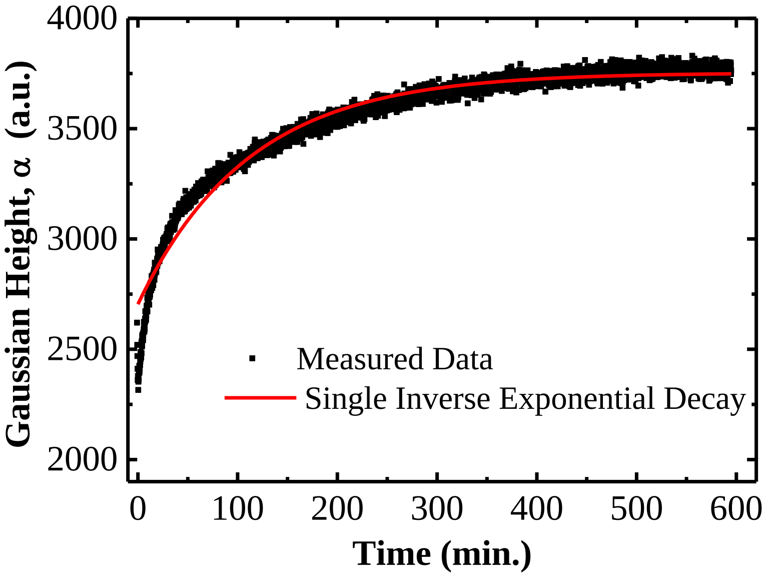

Figure S2: represents the best fit achieved for the time evolution of the Hy-NAA-GIFs signal.

Release profiles for different Hy-NAA-GIFs structures

i) Different flow rates: Figure S3 shows the releasing profiles of the DOX from Hy-NAA-GIFs structures with different flow rates (80 µl/min or 140 µl/min).

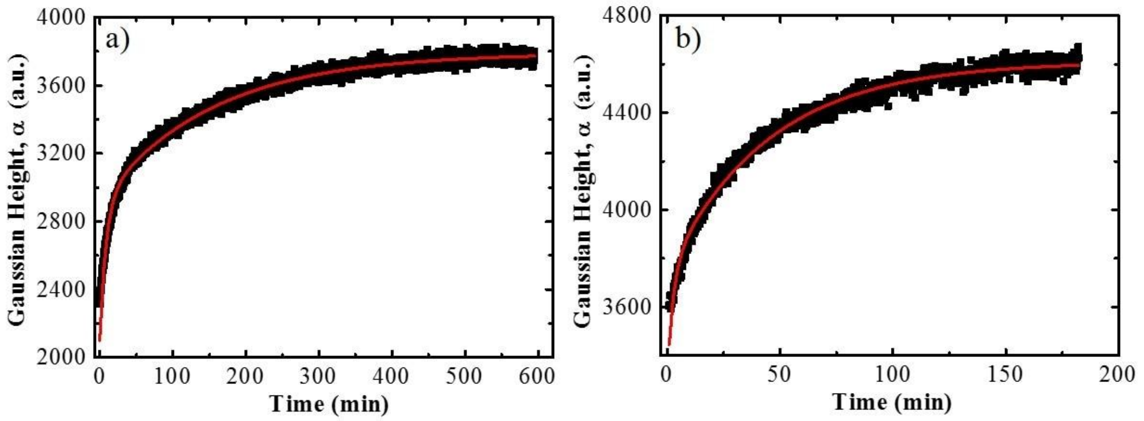

Figure S3: evolution of the parameter α with time showing the release of DOX from Hy-NAA-GIFs for two different flow rates ; a) 80 µl/min, or b) 140 µl/min.

ii) Different pore lengths: Figure S4 shows different releasing behaviors for different lengths of the straight pores of the Hy-NAA-GIFs structures.

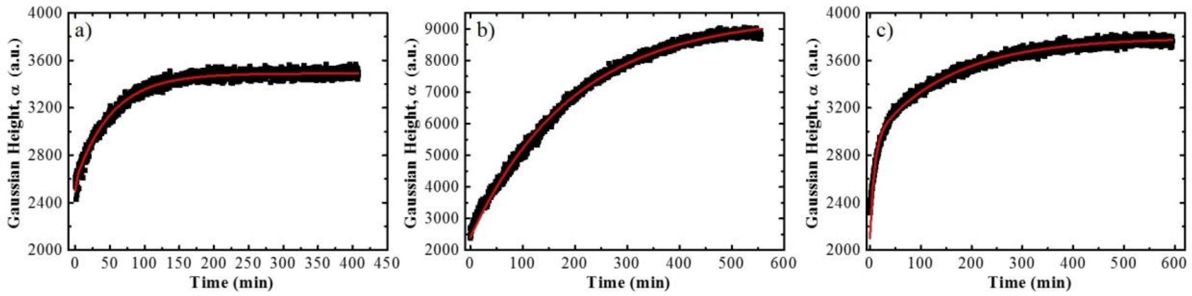

Figure S4: depicts the release of the DOX molecule from Hy-NAA-GIFs for different pore lengths; a) 25 µm, b) 30 µm and c) 84.4 µm.
